# Lived experiences of end-of-life care at home in the UK: a scoping review of qualitative research

**DOI:** 10.3399/BJGP.2023.0349

**Published:** 2024-11-12

**Authors:** Claire Clark, Stephen Fenning, Wendy Haynes, Sarah Clay, Jack Maddicks, Joanna Bowden

**Affiliations:** NHS Fife Specialist Palliative Care Service, Kirkcaldy, Fife.; NHS Fife Specialist Palliative Care Service, Kirkcaldy, Fife.; NHS Fife Library and Knowledge Service, Kirkcaldy, Fife.; NHS Fife Specialist Palliative Care Service, Kirkcaldy, Fife.; Marie Curie Hospice, Edinburgh.; NHS Fife Specialist Palliative Care Service, Kirkcaldy, Fife; School of Medicine, University of St Andrews, Fife.

**Keywords:** caregivers, community care, general practice, home nursing, patient perspectives, palliative care

## Abstract

**Background:**

Home is the preferred place of care and death for most people with advanced illness.

**Aim:**

To examine and describe the published qualitative literature on the lived experiences of dying at home, to characterise the participants and their contexts, and to identify key gaps in knowledge, with a view to informing future research.

**Design and setting:**

A scoping literature review, undertaken in accordance with the PRISMA-ScR guideline, examined studies focusing on the lived experiences of dying at home in the UK.

**Method:**

The online databases CINAHL and MEDLINE were searched with relevant Medical Subject Heading (MeSH) terms and keywords to identify primary qualitative research published between 2010 and 2022, exploring the lived experiences of patients, families, and/or unpaid carers in the UK.

**Results:**

In total, 442 articles were screened, 61 of which underwent full-text review; 13 studies were retained in the final set. All studies explored the experience of bereaved family and/or unpaid carers, and one study interviewed a person who was dying. Where specified, the majority of experiences related to deaths from cancer, many with specialist palliative care team involvement. Included studies yielded a breadth of diverse findings, with the most common subject themes relating to the availability and quality of care, and support for families and carers.

**Conclusion:**

There is limited published evidence exploring the lived experiences of end-of-life care at home and this constrains the extent to which community services can be evidence informed in their design and delivery. More research is needed to examine the first-hand experiences of people who are dying at home, particularly for those with non-cancer conditions and where specialist services are not involved.

## Introduction

Home is the preferred place of care and death for most people with advanced illness, as long as sufficient support is available to them and their families.^1^ Reflecting this, national healthcare strategies now demand that high-quality palliative and end-of-life care should be available to patients in all care settings, including people’s own homes.^2^ In recent years, against the backdrop of the COVID-19 pandemic, there has also been a significant shift towards this care being delivered in the community. Indeed, in Fife (Scotland’s third-largest local authority area by population), there was a 40% increase in the number of deaths at home during the first year of the pandemic.^3^ The authors’ experiences of contributing to the care of the population that is dying at home in Fife during this period were the catalyst for the present study.

GPs and other professionals in primary care teams are recognised as the core providers of palliative care for those dying at home and, as such, are those most likely to be impacted by this increasing demand. It is therefore critical, as Couchman *et al*^4^ argued recently, that primary palliative care is at the centre of research and policy focused on palliative and end-of-life care. This ‘call to arms’ also mirrors the priorities of the Marie Curie and James Lind Alliance Palliative and End-of-Life Care Priority Setting Partnership research,^5^ which highlighted the urgent need to examine the experience of receiving end-of-life care at home and to understand what matters most to patients and families. However, at present, little is known about the existing body of research examining the lived experiences of end-of-life care at home of those who are dying and their caregivers, which, arguably, limits the extent to which local practices and national policy can be evidence informed. To address this, the authors conducted a scoping review that aimed to:

- describe the extent and quality of published qualitative research examining lived experiences of home-based end-of-life care in the UK;
- characterise study participants demographically and clinically, and identify particular populations whose lived experiences are inadequately represented;
- identify key themes from the existing literature and the implications of these findings for practice, as described by the studies’ authors; and
- make recommendations for future research.

**Table:** How this fits in

There is limited published evidence exploring the lived experience of those receiving and supporting end-of-life care at home in the UK, with almost no patient participants. To date, most findings have been generated from interviews with bereaved family carers, who have received specialist palliative care support. More prospective qualitative research is needed to examine the first-hand experiences of patients nearing the end of life at home, including those with non-cancer illness and where specialist palliative care services are not involved.

## Method

A scoping review was undertaken in accordance with the Preferred Reporting Items for Systematic Reviews and Meta-Analyses Protocols Extension for Scoping Reviews (PRISMA-ScR) guidelines.^6^

### Search strategy

A population-concept-context approach was used to define the inclusion criteria. The population comprised adults with any advanced illness who had been identified as nearing the end of life, their family carers, and/or other unpaid carers. Children aged <18 years were excluded as patients, but could be included as carers. The concept was the lived experiences of end-of-life care and support for the dying person, their family, and/or other unpaid carers, including bereavement support. The context was dying, or caring for a person who was dying, in one’s own home or other private residence in the UK. Deaths in care homes, hospices, or hospitals were excluded. The review focused on ‘own-home’ deaths in the UK in order to enable a degree of consistency across the studies and because the authors’ intention was that the review findings should inform future research for this population in Scotland — and, ultimately, inform UK-specific policy and practice, based on evidence of the strengths and limitations of current care and support provision. Eligibility criteria are outlined in Box 1.

**Box 1.** Inclusion and exclusion criteria

**Inclusion criteria** Qualitative or mixed-methods studies with a substantial qualitative component related to experiences of end-of-life care and support Publication date: 2010–2022 Full text available English language Research undertaken exclusively in the UK
**Exclusion criteria** Service evaluations Individual or personal accounts, or case studies Quantitative or mixed-methods studies with limited relevant qualitative content Pre-determined focus on a particular aspect of care (for example, legal needs at end of life) Pre-dominant focus on experience of healthcare professionals Population not identifiably at end-of-life stage of illness End-of-life care in nursing or care homes

Two databases, MEDLINE and CINAHL, were searched in May 2022. The search strategy incorporated keywords relating to the inclusion criteria, and made use of subject terms particular to each database, as shown in Supplementary Box S1. Methodologically, the lack of specific clinical terms for this subject area provided a challenge. It was not possible to exclude results relating to child patients, due to the non-specific way in which age-group subject terms were assigned. Subject terms also tended to focus on the delivery of professional, rather than informal/unpaid care, and the keyword ‘home’ often failed to distinguish between ‘dying at home’ and ‘dying in a [care] home’. It was also not possible to identify meaningful keywords that emphasised lived experience. Many of these limitations were addressed later during the screening process.

**Figure 1.**
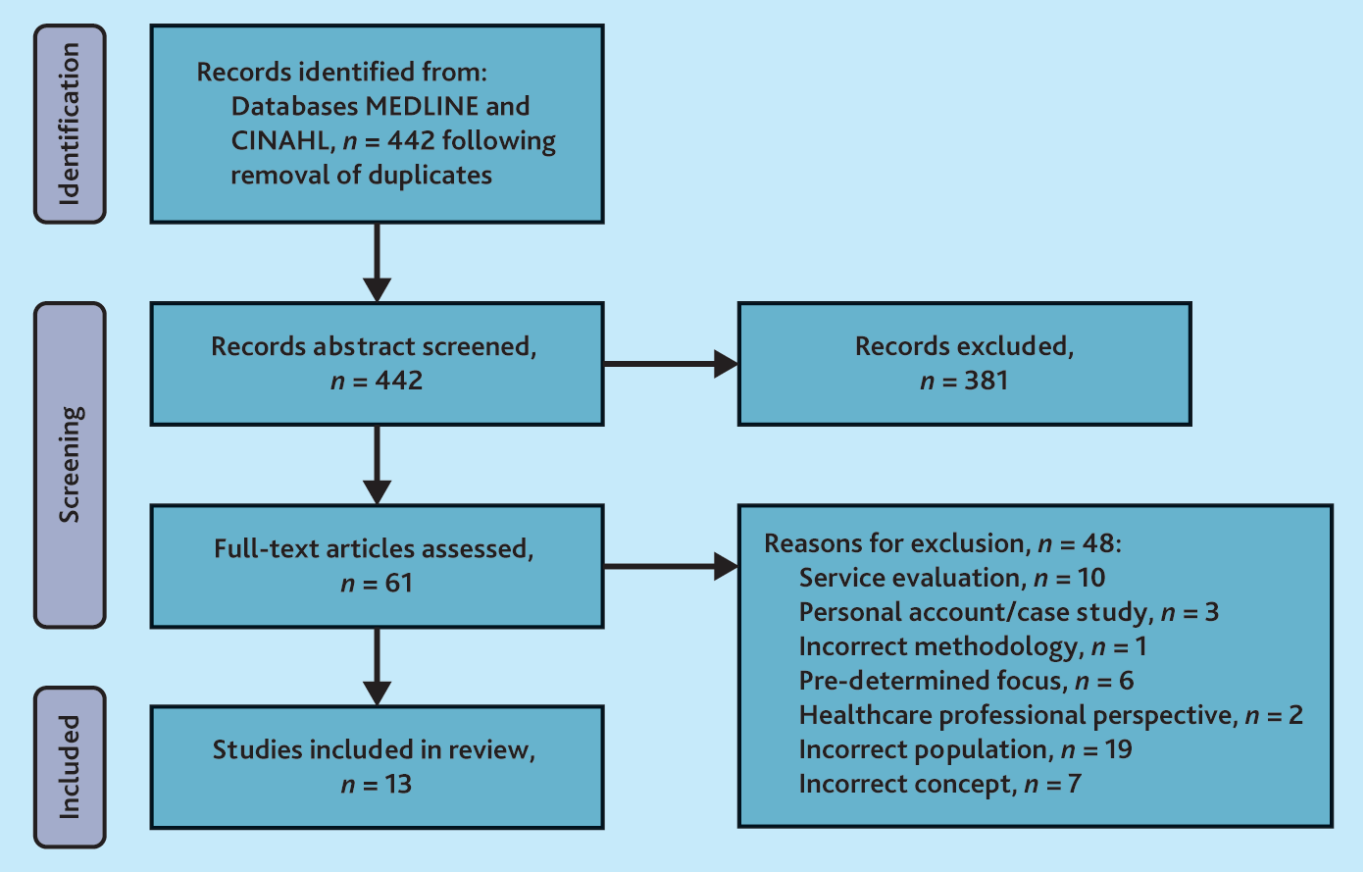
PRISMA flow diagram of the scoping review process.

Titles and abstracts were screened by small groups of two or three study team members and duplicates were removed. Full-text review was undertaken by three authors in pairs. Uncertainty about study eligibility was resolved through discussion with the third researcher. Figure 1 shows the PRISMA flow diagram representation of the literature search process.

### Data extraction

A data extraction form was completed by one author for the included studies. Data fields included: year of publication; UK geographical region; methodological approach; population size, demographics, and clinical characteristics; and key findings, themes, limitations, and implications for practice, as identified by study authors.

Critical appraisal was carried out collaboratively by three authors using the Critical Appraisal Skills Programme (CASP) Checklist for Qualitative Research.^7^ Although not required for data synthesis, critical appraisal enabled an evaluation of each study’s strengths and limitations — in part, to inform the authors’ own group’s future research.

## Results

The literature search yielded a total of 442 results. After the first screening, 61 articles remained, of which 13^8^^–^20 remained following full-text screening. Summary data from all included studies are presented in Supplementary Tables S1 and S2. Supplementary Table S1 details participant and patient demographics, along with authors’ own reported limitations for each study; Supplementary Table S2 documents the results of the authors’ thematic analysis, along with their conclusions and implications for practice.

### Participant and patient population characteristics

In 11^8^^–^18 of the 13 studies, the participants were bereaved caregivers, with interviews taking place 3–24 months after a death. Two studies^19^^,^^20^ interviewed participants (caregivers or patients) when the patients were still alive. Newbury^19^ interviewed caregivers in the weeks leading up to death and again 3 months post-bereavement. Only Percival *et al*^20^ interviewed a (single) patient who was dying, and further patient interviews were deemed impossible because of patient frailty.

Across the 13 included studies, there was wide variation in participant numbers (6–75 participants) and in the reporting of demographic data. Nine studies^8^^,^^9^^,^^11^^–^13^,^^15^^–^18 provided information on participant gender and seven^8^^,^^9^^,^^12^^,^^13^^,^^16^^–^18 stated participant age or age ranges. Only four studies^8^^,^^12^^,^^17^^,^^18^ reported complete ethnicity data; Newbury^19^ commented that participants were *‘all White’* and Lees *et al*^10^ noted that participants were of *‘mainly White female ethnic origin’*. Eight^8^^–^10^,^^12^^,^^15^^,^^16^^,^^18^^,^^19^ of the 13 study populations had both cancer and non-cancer illnesses. Three studies^11^^,^^14^^,^^17^ focused on experiences of those with cancer only, one^13^ focused on dementia only, and one^20^ did not specify the illness profiles of its patient population. Socioeconomic status was not detailed in any study, but four studies^12^^,^^13^^,^^15^^,^^16^ provided general comment on the socioeconomic status of their participants’ geographical area. For example, the contrasting socioeconomic profiles of the two geographical locations.^12^^,^^16^ All included studies were conducted in England and Wales, with four^12^^,^^15^^,^^16^^,^^18^ authored by the same research group.

In four^9^^,^^14^^,^^17^^,^^19^ of the 13 included studies, there was an explicit statement that the patients and families were known to specialist palliative care services. One study^13^ commented that a patient had died in hospice, but did not describe other specialist input. Another study^20^ referred to the involvement of a mix of specialist and non-specialist domiciliary care workers, but not to other professional caregivers. Six studies^10^^–^12^,^^15^^,^^16^^,^^18^ did not specify whether specialist palliative care professionals were involved in care and the majority of authors for these studies did not appear to be directly affiliated with specialist palliative care services.

### Stated aims

Many of the studies had stated aims to explore and/or understand the experiences and views of carers providing end-of-life care at home, including experiences of health and social care services, and factors influencing quality of care. Other specified aims included:

- to illustrate the relevance of carers’ *‘background worries’*;^16^
- to explore the influence of the home environment on the perceived quality of the death;^14^ and
- to examine the role of the domiciliary care workers.^20^

### Methodological approaches

In total, 10 studies^8^^–^11^,^^13^^–^15^,^^17^^,^^19^^,^^20^ reported primary qualitative research, one of which^10^ was derived from a mixed-methods study using the Care of the Dying Evaluation (CODE) questionnaire. Three articles^12^^,^^16^^,^^18^ reported secondary analyses based on interviews described in one of the other included studies.^15^

In total, 11 articles^8^^,^^11^^–^20 drew on data solely from semi-structured or in-depth interviews, one study^9^ employed focus groups and telephone semi-structured interviews, and one study^10^ extracted free-text data from the CODE questionnaire.

The studies’ authors described their data analysis variably, with the majority^9^^–^11^,^^13^^,^^14^^,^^16^^–^18^,^^20^ citing a thematic or narrative analysis with a framework approach. Two studies^12^^,^^15^ specified a cross-sectional thematic analysis, one^19^ a grounded theory approach, and another^8^ utilised a phenomenological interpretive approach.

### Studies’ key findings

The carer role was described frequently as being all consuming. Several studies^10^^,^^13^^,^^20^ identified the themes of competence and/or expertise of paid caregivers and other professionals, highlighting that specific knowledge and skills are needed — and expected — for effective home-based palliative care. Unsurprisingly, the need for continuity of care was a theme in several studies^10^^,^^13^^,^^15^ and was conceptualised by Seamark *et al*^15^ as, not just personal continuity, but also informational and organisational continuity.

Recommendations around the future planning of health and social care services were also common in the studies’ conclusions and included:

- the need for more flexible and responsive domiciliary care services;^18^
- minimising different care staff to improve continuity;^15^ and
- improving bereavement support.^12^

Lees *et al*^10^ made a recommendation for policy (namely, the need to measure the quality of home-based dying) and Ewing *et al*^9^ used their findings to develop the Carer Support Needs Assessment Tool, a validated tool now widely used in the UK, to help professionals support family carers.

Very few articles meaningfully identified or discussed spiritual needs,^8^^,^^9^^,^^17^ but as most studies did not provide detail on the questions posed to participants, it is unclear whether these needs were truly less relevant or whether they were not formally explored.

All studies presented their results as themes. These are presented in Supplementary Table S2 in the authors’ own words, accompanied by a summary of the authors’ conclusions and implications for practice. No secondary analysis of data was undertaken as this was beyond the remit of a scoping review, the aim of which is to synthesise or ‘map’ the available evidence on a topic.^6^

### Studies’ limitations, as described by study authors

All but two studies^9^^,^^12^ discussed their study’s limitations. Five studies^13^^,^^14^^,^^17^^,^^19^^,^^20^ acknowledged that their work was led by specialist palliative care teams with a high proportion of patients with cancer and that findings may not be generalisable to other patient populations. Three articles^13^^,^^17^^,^^18^ commented on a lack of ethnic diversity in their population. Three articles^11^^,^^16^^,^^17^ highlighted the potential for recall bias with findings generated in bereavement.

Other identified limitations included: small study sample;^11^ difficulties in recruiting healthcare professionals^14^ and bereaved family members;^16^ self-selection of participants;^8^^,^^10^^,^^18^ secondary analysis of data;^18^ lack of interpreters precluding the inclusion of non-English speakers^17^ and other *‘potential translator-centred issues’*;^8^ and retrospective design.^15^

### Additional study limitations, as identified by scoping review team

Several limitations, beyond those identified by each study’s authors, were apparent; all of these related to the transparency of reporting. Although most studies used a semi-structured interview method, only two provided any detail on the questions posed to participants who were bereaved: McCaughan *et al*^11^ provided a topic guide within the full text and Pottle *et al*^14^ shared information in an online supplement. In general, there was little detail about the relationship between the study authors and the research participants (where such connections existed); this is important, not only for transparency in reporting but also because the nature of the participant–practitioner/researcher relationship may influence the extent to which negative or challenging real-life experiences are disclosed.^21^

There was also little detail on the demographic characteristics (including ethnicity and socioeconomic status) of study participants and this was reported inconsistently; however, where stated, interviewees were typically bereaved, mostly female family carers who had received support from specialist palliative care services. Given that lived experience can be influenced by many factors, including ethnicity, socioeconomic deprivation, and rurality, the absence of these details constrain the utility of the research findings.

Data on the care and support services involved in delivering each person’s care was also limited, and very few studies described spiritual needs (be they met or unmet), but it was unclear whether these needs were less relevant or had not been formally explored. Experiences receiving minimal or no representation included those of patients living with advanced illness, carers of patients with non-cancer conditions and/or multiple long-term conditions, and patients/carers from minoritised ethnic groups.

### Critical appraisal

Critical appraisal of the studies using the CASP Checklist for Qualitative Research^7^ revealed quality scores ranging from 4 out of 10 to 10 out of 10, with the majority (11 out of 13) given a score of ≥7.^8^^–^11^,^^13^^–^15^,^^17^^–^20 The two most common reasons for the deduction of points were:

- lack of comment on the relationship between researcher and participants; and
- lack of comment on ethical issues (the authors of this review did not deem a brief statement confirming ethics committee approval to be sufficient).

## Discussion

### Summary

This scoping review identified 13 articles, 10 of which reported primary research studies. All studies employed an interview or questionnaire-based methodology. Key themes related to the needs and/or unmet needs of the patient and/or unpaid carer(s), including emotional and practical support. Overall, the included studies were of fair to good quality, but authors frequently failed to acknowledge the relationship between participants and researcher(s). Many studies also lacked detail in the reporting of patient demographics.

### Strengths and limitations

To the authors’ knowledge, this is the first scoping review to examine the published literature on the lived experiences of dying at home in the UK. The main strength of this review is that it focuses on the home setting — a priority area for palliative and end-of-life care research and policy — and provides a previously unavailable oversight of the existing body of research. It has also been undertaken and reported in accordance with the PRISMA-ScR checklist.

The authors focused on research published since 2010 to ensure that the findings captured relatively contemporary experiences of end-of-life care at home. Case studies and opinion pieces were excluded because these do not represent systematically conducted research; in addition, the experience of dying in care homes was excluded because the structure of care and support in these settings differs significantly from that available in private residences.

There are several limitations to this work that should be noted. The search strategy was designed to generate findings relevant to the review authors’ own location (namely, the UK NHS); in so doing however, given the significant differences between health service models in each country within the UK, it is acknowledged that the findings may be less transferrable to other nations. In addition, although some recurring themes emerged across the included studies, a formal thematic synthesis of the included studies’ findings was beyond the remit of a scoping review.

### Comparison with existing literature

Although the authors believe this to be the first formal scoping review of the published literature on the lived experiences of dying at home in the UK, a narrative literature review of international literature published by UK-based researchers in 2015 focused on the perspectives of family carers providing end-of-life care at home.^22^ Morris *et al*^22^ identified 28 studies published between 2000 and 2013, five of which were from the UK, and which utilised a variety of methodologies. As with the review presented here, what emerged strongly in their findings was the importance of support for family and other caregivers, ranging from the practical (personal care) to the clinical (symptom assessment and management), emotional (for one’s own wellbeing as a carer), and informational (around what to expect).

Other published studies have examined specific aspects of end-of-life care, such as legal and financial needs^23^ and the ‘work’ of managing medications.^24^ These articles were identified in the initial literature search undertaken for this scoping review, but were subsequently excluded because the focus of this scoping review was research examining more general lived experiences of end-of-life care at home. However, research on these specific needs is clearly important in contributing to the overall body of evidence on what matters most to patients and families.

The findings of this scoping review resonate strongly with the priorities of the Marie Curie and James Lind Alliance Palliative and End-of-Life Care Priority Setting Partnership research,^5^ specifically their priorities to identify how best to provide support for patients, families, and carers, how best to ensure adequate palliative care training of healthcare staff, and the best ways of providing care in a person’s home.

### Implications for research

Currently, there is limited published evidence exploring the lived experience of people receiving and supporting end-of-life care at home, and the findings of this scoping review highlight several important avenues for future research. Arguably, the most remarkable finding is the relative absence of the patient voice, with only one patient across the 13 studies having been interviewed. More research is needed to examine the first-hand experiences of patients and, although it can be challenging to recruit participants with advanced illness,^25^ it has also been identified that patients are often keen to take part in research and can benefit from the experience.^26^^,^^27^ Furthermore, although carers may be able to describe and advocate for the patient experience, they are, nevertheless, framing experiences through their own prism and cannot provide the authentic patient perspective.

It is also imperative to gain a more complete understanding of the lived experiences of people underrepresented in these studies’ results — namely, those dying from non-cancer illness (including multiple long-term health conditions) and those from minoritised ethnic populations. Similarly, although many researchers may work in, or alongside, specialist palliative care teams, concerted effort must be made to recruit those who do not receive care at home from such teams, as well as those who do. Indeed, in Fife (where the authors of this review work), just one-quarter of the dying population receive support from specialist palliative care services (NHS Fife Specialist Palliative Care Service, unpublished data, 2022).

The paucity of research on lived experiences of end-of-life care at home and the almost universal absence of the patient voice must be addressed as a matter of urgency. There must also be a conscious shift in emphasis towards research into patients receiving primary palliative care as their mainstay of care, not just those receiving specialist palliative care support. This change of focus will likely not only affirm the core role of primary care teams in the delivery of high-quality palliative and end-of-life care in the community, but also illuminate the specific needs of the patients and families under the care of these teams, which will help to inform future policy and practice. It should also be recognised that, at present across the UK, there is already significant unwarranted variation in access to specialist community palliative care services; as such, research into those who are embedded predominantly within specialist services does not always provide real-world validity.

This review’s findings on the underrepresentation of people with multiple long-term health conditions offers further direction for future research — this population has already been identified as an area of interest for the Society of Academic Primary Care’s Palliative Care Special Interest Group.^28^ Future prospective studies should provide accurate and complete demographic data — including reference to participants’ socioeconomic status, ethnicity, and rurality — so that findings can be contextualised. This is particularly important as it is known that such factors can be key determinants of access to care, experiences, and outcomes. In addition, authors should always endeavour to acknowledge the relationship between the participants and researcher(s).

Informed by this scoping review, the authors have since conducted a prospective mixed-methods study of home deaths in Fife, Scotland. Through purposive sampling, they have, with some success, recruited a high proportion of patients into their interview study (compared with the studies identified in this review) and will report full demographic data, including ethnicity and socioeconomic data (based on the Scottish Index of Multiple Deprivation). Furthermore, through close collaboration with colleagues in primary care, they also recruited participants who are not known to specialist palliative care services. These findings will be reported in full separately, but it is hoped that the principles of this scoping review will influence the research of other teams. By having highlighted where gaps in current knowledge exist, this review could add further momentum to the campaign for more research in community palliative and end-of-life care (including that led by primary care) that, in turn, will generate the evidence to guide primary and specialist palliative care services to evolve together and best meet the needs of patients and families at home.
